# The Role of Lipids in Allergic Sensitization: A Systematic Review

**DOI:** 10.3389/fmolb.2022.832330

**Published:** 2022-04-14

**Authors:** Georgina V. Hopkins, Stella Cochrane, David Onion, Lucy C. Fairclough

**Affiliations:** ^1^ School of Life Sciences, The University of Nottingham, Nottingham, United Kingdom; ^2^ SEAC, Unilever, Colworth Science Park, Sharnbrook, United Kingdom

**Keywords:** lipids, allergic sensitization, allergen, protein, NKT cells, dendritic cells, adjuvant, lipid-ligand

## Abstract

**Background:** Immunoglobulin E (IgE)-mediated allergies are increasing in prevalence, with IgE-mediated food allergies currently affecting up to 10% of children and 6% of adults worldwide. The mechanisms underpinning the first phase of IgE-mediated allergy, allergic sensitization, are still not clear. Recently, the potential involvement of lipids in allergic sensitization has been proposed, with reports that they can bind allergenic proteins and act on immune cells to skew to a T helper type 2 (Th2) response.

**Objectives:** The objective of this systematic review is to determine if there is strong evidence for the role of lipids in allergic sensitization.

**Methods:** Nineteen studies were reviewed, ten of which were relevant to lipids in allergic sensitization to food allergens, nine relevant to lipids in aeroallergen sensitization.

**Results:** The results provide strong evidence for the role of lipids in allergies. Intrinsic lipids from allergen sources can interact with allergenic proteins to predominantly enhance but also inhibit allergic sensitization through various mechanisms. Proposed mechanisms included reducing the gastrointestinal degradation of allergenic proteins by altering protein structure, reducing dendritic cell (DC) uptake of allergenic proteins to reduce immune tolerance, regulating Th2 cytokines, activating invariant natural killer T (iNKT) cells through CD1d presentation, and directly acting upon toll-like receptors (TLRs), epithelial cells, keratinocytes, and DCs.

**Conclusion:** The current literature suggests intrinsic lipids are key influencers of allergic sensitization. Further research utilising human relevant *in vitro* models and clinical studies are needed to give a reliable account of the role of lipids in allergic sensitization.

## Introduction

For over 50 years, there has been a substantial, worldwide increase in the prevalence of allergic disease (Pawankar et al., 2013). IgE-mediated allergies are among those increasing in prevalence; globally, IgE-mediated sensitization to environmental allergens (e.g. pollen) affect up to 40% of individuals (Pawankar et al., 2013), and IgE-mediated food sensitization affects up to 10% of children, and 6% of adults (Osborne et al., 2011; Prescott et al., 2013; Lee, 2017; Waserman et al., 2018). In addition, the most recent statistics from the National Health and Nutrition Examination Survey (NHANES) report up to 44.6% of United States children were sensitized to at least one environmental or food allergen source (Salo et al., 2014). Allergic sensitization, the first phase of IgE allergy development, is central to the development of atopic disease. Yet, the underpinning mechanisms of allergic sensitization have not been fully elucidated (van Bilsen et al., 2017). Further research to gain additional insight into these mechanisms is crucial to fully comprehend the pathogenesis of allergic disease, which could consequently drive the development of new treatments.

The mechanisms by which proteins (termed allergenic proteins) from within an allergen source (i.e. peanut) drive allergic sensitization have been explored in more detail compared to the limited research into the role of associated molecules. Indeed, allergenic sources are composed of proteins that are accompanied by other compounds, including carbohydrates and lipids. For instance, the major allergen source, peanut, contains a high abundance of lipid, approximately 49% (Ros and Mataix, 2006). Despite evidence for the high abundance of lipids in various allergen sources, few studies have explored the role of these compounds in allergic sensitization, including their ability to interact with allergenic proteins.

Lipids are small hydrophobic or amphipathic molecules (Fahy et al., 2009) that can be bound or co-delivered with allergenic proteins to the innate immune system. Lipids within an allergen source can be directly associated with allergenic proteins, as some proteins have the capacity to bind lipids through hydrophobic cavities, ionic, or hydrophobic bonds (Jappe et al., 2019). These allergen-bound lipids can be termed protein-lipid complexes. There are several classes of allergenic proteins which have the ability to bind lipids. These allergenic proteins include Bet v 1-like proteins, non-specific Lipid Transfer Proteins (LTPs), 2S albumins, and oleosins (Jappe et al., 2019). These proteins can bind various lipids, depending on their tertiary structure and expression of lipid-ligands, including fatty acids, glycolipids, and phospholipids (Dubiela et al., 2019). This lipid-binding can then result in structural and biochemical changes to the protein, which alters the immune response provoked (Petersen et al., 2014). In contrast to directly binding allergens, lipids from an allergen source can also be co-delivered with the allergenic protein. The lipids can be present in pollen coats of plant allergen sources or in matrices of plant and animal foods. This includes pollen-associated lipid mediators (PALMs) which are bioactive lipids released from the pollen grain, or they can be present in the cell membranes of the allergen source, such as phospholipids (Gilles-Stein et al., 2016). These co-delivered lipids can then interact directly with immune cells to modulate the immune response (Traidl-Hoffmann et al., 2002). It is through allergenic protein-binding and activating immune cells that a variety of intrinsic lipids (lipids within an allergen source), have been shown to influence and promote allergic sensitization.

Indeed, there are numerous papers examining the relationship between intrinsic lipids and allergic sensitization, as discussed in previous reviews (Bublin et al., 2014; Del Moral and Martínez-Naves, 2017). Though this is limited, and there has thus far been no systematic review and synthesis of the available studies. Hence, the aim of this systematic review, believed to be the first on this topic, will appraise all existing literature on the interaction of allergen source-derived lipids with allergenic proteins and cells of the immune system, to influence a Th2 response in IgE-mediated food allergies and aeroallergies. This will contribute to the understanding of the mechanisms underpinning allergic sensitization, as well as provide insight into the different study designs to enable further, much needed research.

## Methods

### Search Strategy

Articles were sought from three databases: PubMed, Web of Science, and EMBASE. One item of grey literature was found *via* Wiley Online Library. Each database was filtered by selecting for articles published in English language as well as excluding reviews.

*See* Supplement 1 for full search terms. The key terms used were as follows: *1*) lipid terms “lipid,” “fatty acid,” “lipid-binding,” “PALM” and *2*) allergy terms “allergy,” “allergies,” “allergen,” “pollen,” “IgE,” “sensitization,” “Th2.” Certain terms were specifically excluded from the search to remove irrelevant results: “pain,” “asthma,” “AHR,” “contact,” “n-3,” “n-6,” “maternal,” “predict,” “prevent,” “dermatitis,” “cross-reactivity,” “profile,” “diagnostic.”

**FIGURE 1:**
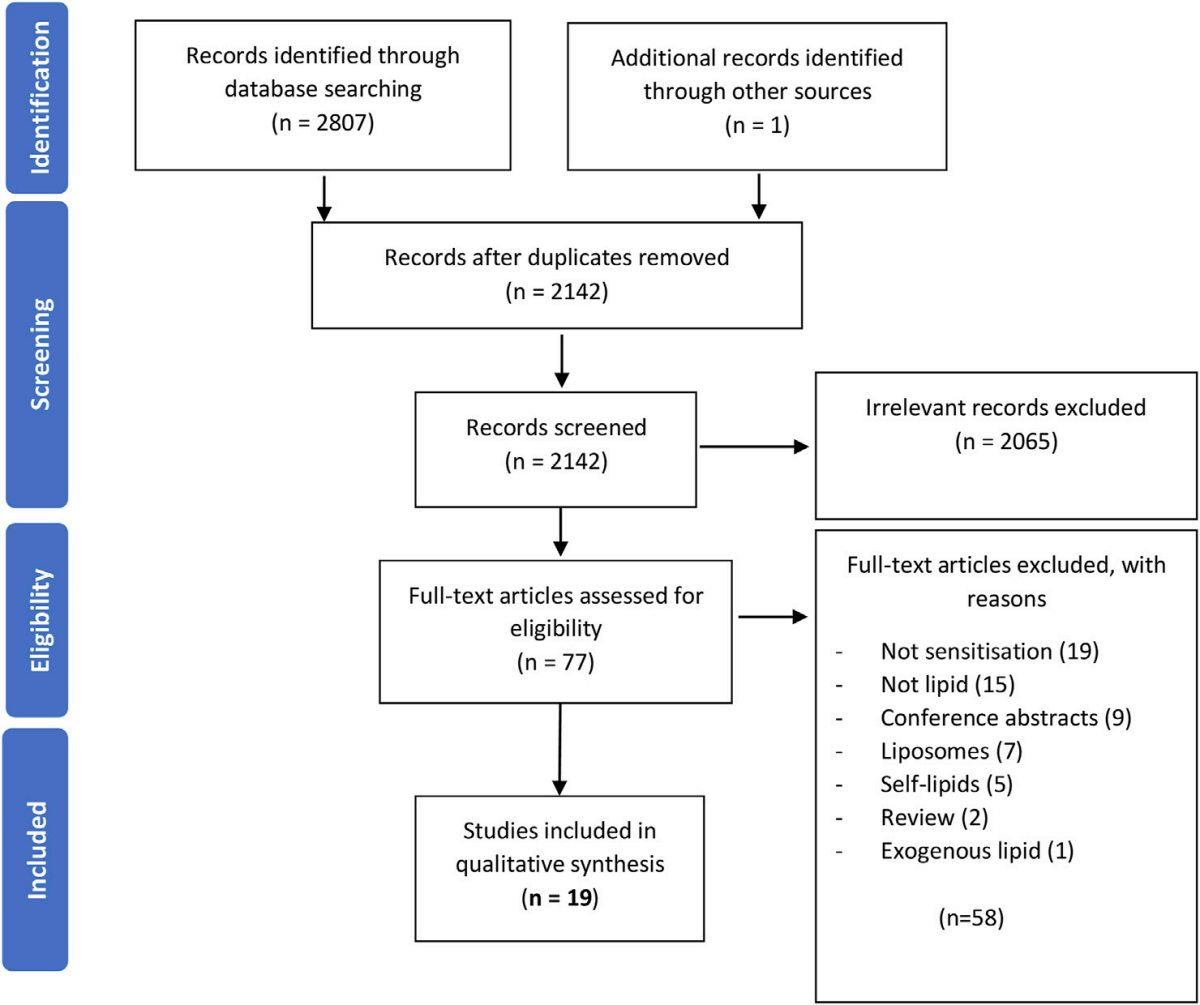
A PRISMA 2009 flow chart detailing the process of study selection. Publications were sought from three databases, duplicates removed, records screened for relevance, full-texts of the remaining articles evaluated for their eligibility, and the remaining studies were grouped into three different categories. This review focuses on the 19 studies investigating the role of lipids in sensitization.

A PRISMA 2009 flow diagram, detailing the process of this systematic review, is shown in Figure 1. The search was conducted on the 18 August 2021 using the terms above, yielding a total of 2,607 articles; PubMed (1806), Web of Science (369), EMBASE (632), and one further article was found using Wiley Online Library. Duplicates were then removed using EndNote software. The remaining titles and abstracts were scanned for relevance to the role of lipids in allergic sensitization. The scanning process was validated by an independent reviewer.

### Inclusion and Exclusion Criteria

Full texts were then assessed for eligibility in the analysis. The full study eligibility criteria is shown in Table 1. In brief, the focus of this review will be on the role of natural lipids found within an allergen source, such as protein-lipid complexes and membrane-bound lipids. The focus is on natural, intrinsic lipids, as the aim of this systematic review is to capture what mechanisms of lipid-influenced allergic sensitization can arise from simply ingesting or inhaling certain allergen sources. Thus, this review excludes any lipids from an exogenous source e.g. microbial lipids, as well as self-lipids (e.g. digestive lipids), as these lipids are not found within an allergen source. The role of omega 3 and omega 6 fatty acids in allergic sensitization were also excluded as there are already many existing systematic reviews, reviews, and position papers within this area (Miles and Calder, 2017; Waidyatillake et al., 2018; Radzikowska et al., 2019; Venter et al., 2019). There were several papers regarding the use of liposomes to capture allergens for drug delivery in allergy treatment. Again, these articles were excluded as they were not natural lipids from an allergen source and did not investigate allergic sensitization.

**TABLE 1:** The inclusion and exclusion criteria used to determine article eligibility for this systematic review.

Inclusion	Exclusion
IgE-mediated food allergy	Non-IgE-mediated allergies
IgE-mediate inhalant allergy	Lipids in asthma
Intrinsic lipids	Lipids in the elicitation phase
Allergic sensitisation	Lipids in the prevention/protection of allergic sensitisation
English language	Non-lipids
Clinical data	n-3 or n-6 fatty acids
Experimental data	Exogenous lipids e.g. Microbial lipids
Healthy subjects	Allergen-encaptured Liposomes for drug delivery
Allergic subjects	Self-lipids e.g. digestive emulsion lipids, cholesterol
Human models	Non-English language publications
Animal models	Conference abstracts
—	Reviews

### Data Extraction

This search resulted in 19 papers which provide data on lipids influencing allergic sensitization. The 19 eligible papers were analysed and the findings synthesised. Data on study design, the subject model, and findings were reviewed.

To quantify the robustness of studies included in this review, the papers were scored based on criteria set in Table 2. Quality assessment scores for each study were determined by the criteria: sample size, the definition of control, representation of the sample, models of allergic sensitization utilised, the robustness of the model, the methods used to prepare lipids, and the characterization of lipid. Scores from each category were summed and divided by the highest possible total score to calculate study quality scores. This scoring system aims to give insight into the designs adopted by current research in this area, where lower-scored studies are not less reliable, but are lacking in characteristics which strengthen the results of the study, such as a small sample size.

**TABLE 2:** Reasoning for study “quality” scores.

Category	Reasoning for scores
Sample size	⁃ Evidence of statistical power calculation to determine the study sample size **(2)**
	⁃ No evidence of power calculation, but sample size was stated **(1)**
	⁃ No power calculation AND sample size was NOT stated **(0)**
Defined controls	⁃ Inclusion of healthy controls **(1)**
	⁃ No healthy controls **(0)**
Representative sample	⁃ 1 point for each of the following: At least 1/3 of each gender **(1)**
	A range of age groups **(1)**
	Inclusion of subjects allergic to the allergen of study **(1)**
	⁃ Unclear sample details **(0)**
	If both human and murine models utilised, the model with the greater representative sample score will be used
Model	⁃ Human (2)
	⁃ Murine **(1)**
	⁃ Unclear **(0)**
	⁃ If both human and murine models are utilised, the study will be awarded the higher mark of **(2)**
Robustness of model	⁃ Animal models: Allergic animals were immunised by intraperitoneal injection/epicutaneous administration **(1)**
	Allergic animals were immunised by intraperitoneal injection/epicutaneous administration AND had specific IgE to allergen OR positive allergen challenge. (2)
	⁃ Human models: Allergic participants were sought from a clinical setting **(1)**
	Allergic participants were sought from a clinical setting AND had a positive skin prick testing to allergen, had specific IgE to allergen, or had a positive allergen challenge **(2)**
	⁃ Unclear allergic subject definitions **(0)**
	⁃ If both human AND murine models were utilised, the model with the greater robustness score will be awarded
Lipid preparation	⁃ The study used commercially sourced lipids (2)
	⁃ The study performed extraction of the lipid from the allergen source **(1)**
	⁃ The study performed extraction of the lipid from the allergen source AND further purification of lipid (2)
	⁃ The study used commercially sourced lipids AND extracted/purified lipids from an allergen source (2)
	⁃ Unclear **(0)**
Lipid characterisation	⁃ The exact lipids responsible for the outcomes were defined e.g. polar lipids, fatty acids, phospholipids. (2)
	⁃ The lipids responsible for the outcome were not well defined e.g. simply “pollen lipids” or “aqueous pollen extract” or “peanut lipids.” **(1)**
	⁃ The lipids used were not defined e.g. “lipids” **(0)**
Overall quality score	The combined score for the categories above divided by the highest possible score of 14

Quality assessment scores for each study were determined by the criteria: sample size, the definition of control, representation of the sample, models of allergic sensitization utilised, the robustness of the model, the methods used to prepare lipids, and the characterization of lipid. The overall score was calculated by the sum of each category, divided by the highest possible total score.

## Results

The 19 relevant papers included in this systematic review, (Agea et al., 2005; Dearman et al., 2007; Gilles et al., 2009; Gutermuth et al., 2007; Gilles et al., 2010; Jyonouchi et al., 2011; Mirotti et al., 2013; Oeder et al., 2015; Angelina et al., 2016; Bansal et al., 2016; Satitsuksanoa et al., 2016; Abos Gracia et al., 2017; Tordesillas et al., 2017; Hufnagl et al., 2018; Pablos-Tanarro et al., 2018; Palladino et al., 2018; González Roldán et al., 2019; Finkina et al., 2020; Meng et al., 2020) which are directly relevant to the role of lipids in allergic sensitization, illustrate the majority of studies were conducted within the last decade. All papers were published within the last 15 years, with 58% published in the last 5 years (2016–2021).

Of the 19 studies reporting a relationship between lipids and allergic sensitization, 16 solely report lipids enhance allergic sensitization (Agea et al., 2005; Dearman et al., 2007; Gilles et al., 2009; Gilles et al., 2010; Jyonouchi et al., 2011; Mirotti et al., 2013; Oeder et al., 2015; Angelina et al., 2016; Bansal et al., 2016; Satitsuksanoa et al., 2016; Abos Gracia et al., 2017; Tordesillas et al., 2017; Pablos-Tanarro et al., 2018; González Roldán et al., 2019; Finkina et al., 2020; Meng et al., 2020), two studies report lipids can both enhance and inhibit allergic sensitization (Gutermuth et al., 2007; Palladino et al., 2018), and one study observed lipids inhibited allergic sensitization (Hufnagl et al., 2018).

The results of the systematic review have been split into two sections; lipids associated to food allergens and lipids associated to aeroallergens.

### Intrinsic Lipids in Driving Food Allergies

Of the 19 articles, 10 of these discuss the role of lipids in allergic sensitization to food allergenic proteins (Dearman et al., 2007; Jyonouchi et al., 2011; Mirotti et al., 2013; Angelina et al., 2016; Tordesillas et al., 2017; Hufnagl et al., 2018; Pablos-Tanarro et al., 2018; Palladino et al., 2018; Finkina et al., 2020; Meng et al., 2020). Nine out of the 10 papers found lipids can shift or enhance allergic sensitization of food allergenic proteins (Dearman et al., 2007; Jyonouchi et al., 2011; Mirotti et al., 2013; Angelina et al., 2016; Tordesillas et al., 2017; Pablos-Tanarro et al., 2018; Palladino et al., 2018; Finkina et al., 2020; Meng et al., 2020). Table 3 summaries the methods and outcomes for these food allergy studies (N.B. Only factors of the articles that were relevant to the role of lipids in allergic sensitization were included in the table).

**TABLE 3:** A summary of the primary articles discussed, relevant to lipids in food allergies.

First author [Ref]	Year	Allergen(s)	Lipid(s)	Cells Responding	Model	Method	Outcome	Effect on allergic sensitisation
Angelina	2016	Sin a 2 and Ara h 1	Phospholipids, peanut and mustard lipids	Dendritic cells	Human	Human sera from patients allergic to mustard or peanuts were collected. Allergen-lipid binding was assessed by SDS-PAGE and spectroscopic binding assays. The ability of dendritic cells (DCs) to capture and uptake peanut/mustard allergens, with or without lipids, was measured by flow cytometry and confocal microscopy, along with cytokine levels	Sin a 2 and Ara h 1 bound phosphatidylglycerol acid and intrinsic lipids, resulting in resistance to gastrointestinal digestion, reduced uptake by DCs, retained Immunoglobulin E (IgE) reactivity of allergen, increased IL-1B levels and increased protection from microsomal degradation	Enhances allergic sensitisation
Palladino	2018	Ara h 1, Ara h 2	Peanut lipids	Keratinocytes	Human	Human keratinocytes were exposed to peanut lipids with or without the major peanut allergens, Ara h 1 or Ara h 2 and their cytokine release measured by enzyme-linked immunosorbent assay (ELISA)	Peanut lipids with or without allergen stimulated human keratinocytes to increase production of GM-CSF. Peanut lipids alone increased IL-10 secretion from keratinocytes. Whereas peanut lipids with allergen inhibited IL-10 secretion	Enhances and inhibits allergic sensitisation
Dearman	2007	Ber e 1	Brazil nut lipids	N/A	Murine	Female BALB/c mice immunised with Ber e 1, combined with and without natural brazil nut lipids. Serum samples were analysed for Ber e 1-specific IgE and IgG in assays	Ber e 1 with total lipid fraction produced significant adjuvant effects on Immunoglobulin G (IgG) and IgE. Natural Ber e 1 containing endogenous lipids also produced IgG and IgE antibody	Enhances allergic sensitisation
Mirotti	2013	Ber e 1	Brazil nut lipids	iNKT cells	Murine and human	Female BALB/c mice were sensitised to Ber e 1 and specific lipid fractions, followed by IgE measurements by ELISA and passive cutaneous anaphylaxis (PCA). Lipid-binding of Ber e 1 was measured using fluorescent probes and NMR. *In vitro* production of IL-4 was measured by flow cytometry and ELISA.	Lipid fraction (lipid C) interacted with Ber e 1 *via* a lipid-binding site to induce Ber-specific IgE. iNKT-deficient mice produced lower levels of IgE than wild type. *In vitro*, Ber/lipid C-stimulated murine iNKT cells produced IL-4 but not IFN-γ in a CD1d dose-dependent manner	Enhances allergic sensitisation
Tordesillas	2017	Pru p 3	Pru p 3 lipid-ligand: Phytosphingosine tail	iNKT cells, epithelial cells, and MoDCS	Murine and human	*In vitro* cultures of human moDCs, PBMC, epithelial and murine DN32.D3, and invariant natural killer T (iNKT) hybridoma cell lines were incubated with the Pru p 3 lipid-ligand extracted from peach peel extract. Cells were assessed for maturation, IgE production, and cytokine production	The lipid-ligand of Pru p 3 induced the maturation of moDCs. It induced higher levels of IgE than Pru p 3 alone. The immunological capacity of the Pru p 3 ligand was mediated by CD1d and was able to activate murine iNKTs	Enhances allergic sensitisation
Hufnagl	2018	Milk lipocalin Bos d 5	Retinoic acid	T cell	Human	*In vitro* and in *silico* retinoic acid (RA)-Bos d 5 binding assays were performed. PBMCs stimulated with Bos d 5 and T cells were assessed by flow cytometry and their cytokine release	Bos d 5 has high binding affinity to retinoic. RA-bound Bos d 5 decreased CD3^+^CD4^+^ cell types and supressed IL-10, IL-13 and IFN-y production. This reduced the immunogenicity of Bos d 5 and its allergenicity	Inhibits allergic sensitisation
Jyonouchi	2011	Milk and egg allergens	Cow’s milk-sphingomyelin, hen’s egg-ceramide	iNKT cells	Human	PBMCs from children with cow’s milk or hen’s egg allergy, and healthy controls were incubated with α-GalCer, cow’s milk–sphingomyelin, or hen’s egg–ceramide. iNKTs were quantified, and their cytokine production and proliferation were assessed. Human CD1d tetramers loaded with milk-sphingomyelin or egg-ceramide were used to determine food-sphingolipid binding to the iNKT-T cell receptor (TCR)	Milk-sphingomyelin, but not egg-ceramide, engaged the iNKT-TCR and induced iNKT proliferation and T-helper 2 (Th2)-type IL-4 secretion. Children with food allergy had significantly fewer peripheral blood iNKTs which exhibited a greater Th2 response to α-GalCer and milk sphingomyelin compared to iNKTs of healthy controls	Enhances allergic sensitisation
Finkina	2020	Len c 3	Fatty acids: oleic C18:1 (OLE), lauric acid C12:0 (LAU), stearic C18:0 (STE), and behenic C22:0 (BEH)	N/A	Human	Circular dichroism spectroscopy was used to assess the influence of the selected	The binding of OLE, LAU, and STE all reduced the rate of Len c 3 gastric degradation, apart from BEH. STE and OLE increased thermostability of Len c 3, whereas LAU and BEH had only a slight protective effect on the secondary structure. No lipid-ligand affected IgE binding capacity of Len c 3	Enhances allergic sensitisation
						Fatty acids on thermostability of rLen c 3. Gastrointestinal degradation of Len c 3 was simulated and characterised by RP-HPLC and SDS-PAGE. Allergen-specific IgE ELISAs were conducted to determine IgE binding abilities of Len c 3 with lipid-ligands		
Meng	2020	α-lactalbumin (BLA) and β-lactoglobulin (BLG)	C18 unsaturated fatty acid (UFA)	N/A	Human	The secondary and tertiary structures of BLA and BLG after treatment with C18 UFAs were characterized by circular dichroism (CD) spectroscopy, ultraviolet (UV) absorption spectroscopy, and ANS fluorescence spectroscopy. Potential allergenicity was determined by Inhibition IgE ELISAs with milk-allergic patients’ sera	The binding of whey allergens to C18 UFAs resulted in the unfolding of BLA and BLG protein structures. This change in structure resulted in the enhanced IgE binding ability of BLA and BLG.	Enhances allergic sensitisation
Pablos-Tanarro	2018	Egg	Egg yolk lipids	Intestinal epithelial cells, Dendritic cells	Murine and human	Female BALB/c mice were orally sensitised to egg white and egg yolk with/without adjuvant or intraperitoneally without adjuvant. *In vitro* assays assessed human epithelial and dendritic cell functions	Egg yolk produced Th2-biasing effects through the upregulation of intestinal IL-33 expression. Egg yolk also favoured Th2 polarisation during DC presentation of allergens to T cells	Enhances allergic sensitisation

Key details of each food allergy study are presented, along with whether the study provides evidence for the role of lipids driving or inhibiting allergic sensitization.

These 10 studies identify three main mechanisms of food-derived lipids influencing allergic sensitization: the activation of iNKT cells through CD1d molecules, direct activation of immune cells, and the induction of conformational changes to allergenic proteins. The evidence for these mechanisms will now presented in further detail.

### CD1d-Restricted iNKT Cell Activation

Four of the 10 food allergy studies report lipid presentation by CD1d molecules (Dearman et al., 2007; Jyonouchi et al., 2011; Mirotti et al., 2013; Tordesillas et al., 2017), with three of these also reporting the activation of iNKT cells (Jyonouchi et al., 2011; Mirotti et al., 2013; Tordesillas et al., 2017).

Intrinsic lipids can be delivered to the immune system bound to allergenic proteins. This CD1d-iNKT cell mechanism is evident in the case of the lipid-ligand of Pru p 3 (from peach), in particular its lipid phytosphingosine tail, which was shown to activate murine-derived iNKT cells (determined by IL-2 secretion), through its lipid-ligand presentation on CD1d molecules (Tordesillas et al., 2017). Another study found the allergen protein, Ber e 1, failed to induce IgE production in sensitised mice when administered without its lipid fraction (Dearman et al., 2007). When the lipid fraction was present, it acted as an adjuvant to IgE production. It was suggested the adjuvant activity of the lipid fraction could be due to its ligation of CD1d molecules (Dearman et al., 2007). A subsequent study of Ber e 1 sensitization found the lipid fraction, named “lipid C,” induced the production of the Th2 cytokine IL-4 from iNKT cells to shift to allergic sensitization. They also found Ber e 1 can bind “lipid C” *via* a hydrophobic pocket, allowing the protein-lipid complex to ligate CD1d molecules (Mirotti et al., 2013). One study investigated milk and egg lipids, sphingomyelin and ceramide, respectively, in allergic sensitization (Jyonouchi et al., 2011). They established milk-sphingomyelin, but not egg-ceramide, can induce Th2-skewing of iNKT cells by presentation on human CD1d molecules. Unlike the aforementioned studies, this study also evaluated iNKT cell populations, revealing children with milk allergy had fewer iNKT cell numbers, but greater Th2 responses to milk-sphingomyelin than the iNKT cells of non-milk allergy controls.

Overall, all four studies report some lipids do promote allergic sensitization through CD1d presentation on DCs and subsequent activation of CD1d-restricted iNKT cells. The quality of these studies were assessed and the calculated scores were similar, with two out of four studies scoring 0.86 (Mirotti et al., 2013; Tordesillas et al., 2017), one study scored 0.79 (Jyonouchi et al., 2011), and the final study was awarded a lower score of 0.64 (Dearman et al., 2007) (Table 4). Notably, this study used murine models only, which contributed to its lower score.

**TABLE 4:** A summary of the quality of each food allergy study included in this systematic review.

First author (Year) [reference]	Sample quality			Methodological quality				Overall quality score (n/1)
	Sample size (n/2)	Defined controls (n/1)	Representative sample (n/3)	Model (n/2)	Robustness of model (n/2)	Lipid preparation (n/2)	Lipid characterisation (n/2)	
Angelina (2016)	Unknown (0)	Yes (1)	Unknown (0)	Human (2)	Allergic samples sought from allergy unit within a hospital (1)	Phospholipids commercially sought and passed through an extruder	Phospholipids	0.54
						Mustard/peanut lipids extracted from source and purified. (2)	Phosphatidylglycerol, PhosphatidylcholineMustard/peanut lipids. (1.5)	
Palladino et al. (2018)	Unclear, at least 3 (1)	Yes (1)	Unknown (0)	Human (2)	Unknown (0)	Peanut lipids extracted and purified. (2)	Peanut lipids (1)	0.50
Dearman (2007)	“groups of five mice” (1)	Yes (1)	Female BALB/c mice, 8–12 weeks old, allergic subjects (2)	Murine (1)	Mice sensitised by intraperitoneal (i.p.) injection of allergen and total IgE (not allergen specific) measured (1)	Total lipids extracted from Brazil nuts and purified. Lipids were then separated into classes by chromatography. (2)	Brazil nut b-sitosterol, total lipid fraction, sterols, free fatty acids, polar lipids (2)	0.71
Mirotti (2013)	Unknown mice numbers, four humans. (1)	Yes (1)	Female BALB/c mice, 8–12 weeks old, allergic subjects	Human and Murine (2)	Mice sensitised by ip injection of allergen and total IgE (not specific) measured	Lipids extracted and purified from brazil nut. (2)	Brazil nut “Lipid C”: mainly triglycerides, sterylglycosides, Phosphatidylethanolamine, PC, phosphatidic acid, and a sulphonated	0.86
			Unknown human participant characteristics. (2)		Human allergic subjects selected by positive skin prick tests to brazil nut/walnut/peanut (2)		di-galacto lipid (2)	
Tordesillas (2017)	Unclear, at least 8 (1)	Yes (1)	Female 6–8 weeks old C3H/HeOuJ mice, unknown human subject details, Allergic subjects (2)	Human and murine (2)	Mice sensitised by epicutaneous administration and specific IgE measured	Lipid-ligand extracted from peach peel and separated by chromatography. (2)	Pru p 3 lipid-ligand (phytosphingosine tail) (2)	0.86
					Unknown human sample details. (2)			
Hufnagl (2018)	29 allergic, (1)	Yes (1)	Children only, allergic subject included (1)	Human (2)	Allergic/healthy participants defined by positive/negative oral allergen challenge to milk, respectively. (2)	Lipid sought commercially. (2)	All-trans retinoic acid (2)	0.79
Jyonouchi (2011)	27 (1)	Yes (1)	23 males and four females, children only, allergic subjects included (1)	Human (2)	Allergic participants had a positive skin prick test and/or presence of specific IgE, positive food challenge and clinical stability on a diet excluding milk and/or egg. (2)	Lipids commercially sought. (2)	Cow’s milk–sphingomyelin, or hen’s egg–ceramide (2)	0.79
Finkina (2020)	10 human sera samples (1)	Yes (1)	Unknown (0)	Human (2)	Allergic samples obtained from a clinical diagnostic centre at a research institute. (1)	Lipids commercially sought. ((2)	Fatty acids: oleic C18:1 (OLE), lauric acid C12:0 (LAU), stearic C18:0 (STE), and behenic C22:0 (BEH) (2)	0.64
Meng (2020)	10 human sera samples (1)	Yes (1)	Seven male, three females, and a range of age groups. Allergic subjects included. (2)	Human (2)	Allergic samples sought from patients at a hospital (1)	Lipids commercially sought. (2)	C18 unsaturated fatty acids from: oleic acid (OA), linoleic acid (LA), c9, t11-conjugated linoleic acid (CLA), α-linolenic acid (ALA), and γ-linolenic acid (GLA).(2)	0.79
Pablos-Tanarro (2018)	Unclear, at least four humans (1)	Yes (1)	Female 6-week old BALC/c mice, allergic subjects (2)	Human and murine (2)	Oral or ip. injection sensitisation and allergen-specific IgE measurement	Egg yolk separated from egg white. (1)	Egg yolk lipids (1)	0.71
					Unknown human sample details. (2)			

Studies were scored out of one for sample quality and methodological quality. Only aspects of each study relevant to the role of lipids in allergic sensitization were scored.

### Lipids Activate Immune Cells

Five of the 10 food allergy studies investigated the role of lipids in directly activating immune cells (Dearman et al., 2007; Angelina et al., 2016; Tordesillas et al., 2017; Pablos-Tanarro et al., 2018; Palladino et al., 2018).

Three of the five studies found lipids do enhance allergic sensitisation. One such study established the mustard seed and peanut allergen proteins, Sin a 2 and Ara h 1 respectively, accompanied by lipids derived from mustard and peanuts, reduced human monocyte-derived dendritic cell (hmoDC) allergenic protein uptake (Angelina et al., 2016). Reduced protein uptake favours a Th2 reaction, whereas higher doses of protein uptake results in tolerance (Wisniewski et al., 2013). Another study discovered egg yolk lipids acted as a Th2-biasing adjuvant to egg white through the upregulation of intestinal IL-33 by epithelial cells *in vitro*, which is crucial for DC activation and Th2 priming (Pablos-Tanarro et al., 2018). In addition to providing CD1d-iNKT activation evidence above, one study of the lipid-ligand of Pru p 3 (from peach) also established the lipid directly activated DCs as it matured human monocyte-derived DCs (Tordesillas et al., 2017).

One of the five studies revealed evidence for and against lipids enhancing allergic sensitization (Palladino et al., 2018). This study, related to peanut sensitization, found the administration of peanut lipids alone resulted in increased production of the anti-inflammatory cytokine, IL-10, from keratinocytes, thus inhibiting a Th2-type response. Whereas, peanut lipids delivered with the peanut allergenic protein inhibited IL-10 production.

Another nut allergen source study, also mentioned previously, found the allergenic protein, Ber e 1, failed to induce IgE production in sensitised mice when administered without its lipid fraction. It was only when the lipid fraction of the Brazil nut was present, the lipid acted as an adjuvant to IgE production. The total lipid fraction of the Brazil nut, including its composite sterols and polar lipids, all had marked adjuvant effects on IgE production. However, b-sitosterol and glycolipid-rich fractions had negligible impact on IgE production (Dearman et al., 2007).

As shown in Table 4, the four studies solely stating lipids enhance allergic sensitization received quality scores of 0.54 (Angelina et al., 2016), 0.64 (Dearman et al., 2007), 0.71 (Pablos-Tanarro et al., 2018) and 0.86 (Tordesillas et al., 2017).The study stating lipids may inhibit allergic sensitization received the lowest score of all the included food allergy studies, receiving a score of 0.50 (Palladino et al., 2018). This study mainly lost points due to lack of reporting sample characteristics such as sample size, gender, age, and how they defined their allergic and healthy cohort.

### Lipids Induce Conformational Changes of Allergens

Four out of the eight food allergen studies measured the influence of lipids on the structure of their associated allergenic proteins (Angelina et al., 2016; Hufnagl et al., 2018; Finkina et al., 2020; Meng et al., 2020).

The digestibility of food proteins can determine whether the protein is tolerated or becomes a sensitizing agent. High resistance to digestion in the gastrointestinal tract has been shown to increase the sensitization capacity of proteins (Pali-Schöll et al., 2018). Three of these studies suggest protein-lipid binding can influence allergenic protein structure to alter digestion, and thus alter the sensitization capacity of the allergenic protein (Angelina et al., 2016; Finkina et al., 2020; Meng et al., 2020). One study found that, in addition to lipids intrinsic to an allergen source, the peanut allergenic proteins, Sin a 2 and Ara h 1, can also interact with membrane-bound lipids (lipids derived from the cell membrane of the allergen source), such as phospholipids (Angelina et al., 2016). This study, previously mentioned above as evidence for the direct activation of DCs, highlights that Sin a 2 and Ara h 1 can bind phosphatidylglycerol (PG) vesicles, reducing their gastrointestinal degradation (Angelina et al., 2016). Furthermore, the ability for proteins to bind PG vesicles was dependent on the pH conditions; at pH 2.0, the phospholipids increased α-helix in Sin a 2 and β-sheet in Ara h 1, thus enhancing the content of allergenic protein secondary structure. In contrast, this was not the case for the mustard seed allergenic protein, Sin a 3, which is structurally different to peanut allergenic proteins, which highlighted it’s structure and digestion was not affected by the presence of PG vesicles (Angelina et al., 2016).

Another study focused on the plant lipid transfer protein (LTP), Len c 3 (Finkina et al., 2020). It has been established that legumes contain a high level of lipids, composing mostly of unsaturated fatty acids (Grela and Günter, 1995). The lentil allergenic protein, Len c 3, is highly stable to digestion. This study found Len c three binding of the unsaturated fatty acids: oleic acid (OLE), lauric acid (LAU), and stearic acid (STE), all reduced the rate of Len c three gastric degradation, apart from behenic acid (BEH) which did not alter degradation. Furthermore, OLE reduced Len c three degradation to 55% after 24 h of simulated digestion, compared to 100% of Len c 3 degraded after 24 h with no ligand. STE and OLE increased thermostability of Len c 3, while increasing the content of α-helices. Whereas, LAU and BEH only had a slight protective effect on the secondary structure. Despite these conformational changes, no lipid-ligand increased the IgE binding capacity of Len c 3.

In contrast, another study found protein-lipid binding did enhance the IgE-binding abilities of both whey proteins, α-lactalbumin (BLA) and β-lactoglobulin (BLG) (Meng et al., 2020). Whey proteins derived from cow’s milk are widely used in the food industry due to their ability to emulsify, foam, and gelatinise food products (Lucey et al., 2017). These whey proteins also constitute the common allergenic proteins, α-lactalbumin (BLA) and β-lactoglobulin (BLG). Thus, the ability to reduce their allergenicity would be profitable to the food industry. The linear and conformational epitopes of proteins contribute towards the allergenicity of the allergen (Hochwallner et al., 2010). This study found BLA and BLG can bind C18 unsaturated fatty acids (UFA) to form protein-ligand complexes (Meng et al., 2020). This binding to the fatty acid resulted in the structural unfolding of BLG, where C18 UFA treatment induced a transition from a β-sheet to a random coil. Furthermore, BLA treatment with C18 UFA resulted in changes to tertiary structure. Therefore, this study suggests protein-lipid binding can alter allergenic protein structure which alters the allergenicity of the milk allergens.

In contrast, one study found intrinsic lipids do not alter allergenic protein structure and further stated they do not drive allergic sensitization (Hufnagl et al., 2018). Retinoic acid, found in cow’s milk, had a high binding affinity for the common milk allergen protein, Bos d 5. This lipid did not alter the conformation of Bos d 5, and so not surprisingly did not alter its allergenicity or IgE binding in allergic children. Furthermore, the protein-lipid complexes supressed CD3^+^ CD4^+^ cell numbers which indicates an immunosuppressive effect on this population, which is pivotal in allergy induction.

Overall, three out of the four studies (Angelina et al., 2016; Finkina et al., 2020; Meng et al., 2020) found lipids induced conformational changes of allergenic proteins which influenced allergic sensitisation, with one study suggesting that some lipids do not alter protein structure and thus allergenicity (Hufnagl et al., 2018). These studies highlight that different lipids, even those from the same class, have different effects on the structure of allergenic proteins. Furthermore, the quality assessment for these studies (Table 4) was mixed, with scores of 0.54 (Angelina et al., 2016), 0.64 (Finkina et al., 2020), and 0.79 (Hufnagl et al., 2018; Meng et al., 2020). Notably, one of the highest scoring studies stated retinoic acid does not promote allergic sensitization to milk allergens (Hufnagl et al., 2018), gaining points as it is one of the only studies of the review which utilised a large, well-defined cohort of human patients.

### Summary of Lipids in Food Allergies

Seven out of the 10 papers discussed how lipid-allergenic protein binding influences allergic sensitization (Dearman et al., 2007; Mirotti et al., 2013; Angelina et al., 2016; Tordesillas et al., 2017; Hufnagl et al., 2018; Finkina et al., 2020; Meng et al., 2020), with one also studying membrane-bound lipids (Angelina et al., 2016). The final three papers studied the impact of lipids directly on immune cells (Jyonouchi et al., 2011; Hufnagl et al., 2018; Palladino et al., 2018). The proposed mechanisms of lipids promoting allergic sensitization include activating iNKT cells through CD1d presentation, resulting in the upregulation of Th2 cytokines. Lipids also directly activate immune cells such as DCs to modulate activation and its allergenic protein uptake. There was also evidence for lipids inducing conformational changes to the protein to result in reduced gastrointestinal degradation of the protein, which shifts towards a Th2 response. Although, two out of the 10 papers on food allergies provided limited data showing that lipids can supress allergic sensitization to allergens when the lipids were delivered without the allergenic protein (Palladino et al., 2018) and by supressing CD3^+^CD4^+^ T cell populations (Hufnagl et al., 2018).

### Intrinsic Lipids in Aeroallergies

Nine out of the total 19 studies examined the role of intrinsic lipids in allergic sensitization to aeroallergens (Agea et al., 2005; Gutermuth et al., 2007; Gilles et al., 2009; Gilles et al., 2010; Oeder et al., 2015; Bansal et al., 2016; Satitsuksanoa et al., 2016; Abos Gracia et al., 2017; González Roldán et al., 2019). All nine studies reported the lipids do enhance allergic sensitization (Agea et al., 2005; Gutermuth et al., 2007; Gilles et al., 2009; Gilles et al., 2010; Oeder et al., 2015; Bansal et al., 2016; Satitsuksanoa et al., 2016; Abos Gracia et al., 2017; González Roldán et al., 2019). Although, one of these studies highlight lipids can also inhibit a Th2 response (Gutermuth et al., 2007). Table 5 outlines the methods and outcomes of these studies.

**TABLE 5:** A summary of the primary articles discussed, relevant to lipids in aeroallergies.

First author [Ref]	Year	Allergen(s)	Lipid(s)	Cells Responding	Model	Method	Outcome	Effect on allergic sensitisation
Agea	2005	Cypress pollen	PALMs: phosphatidylc-holine (PC), phosphatidyle-thanolamine (PE)	CD4^+^ T Cells, Dendritic cells	Human	T cell lines from cypress pollen-sensitive individuals were pulsed with cypress pollen lipids and cytokine responses were measured by ELISA. DC capture of pollen grains were assessed in the presence of anti-CD1d and anti-CD1a and analysed by confocal imaging	PC and PE pollen lipids stimulated the proliferation of T cells from cypress-sensitive subjects and required CD1a+ and CD1d+ antigen presenting cells for lipid recognition. The responding T cells secreted both IL-4 and IFN-y	Enhances allergic sensitisation
Abos Gracia	2017	Olea	Olive pollen lipids (polar lipids, diaglycerolds, triaglycerols, free fatty acids)	iNKT cells, macrophages, and dendritic cells	Human	Invariant natural killer T (iNKT) cells, macrophages, and DCs were obtained from healthy blood donors, using flow cytometry to determine phenotype and cytotoxic killing assay to determine iNKT cell activation	iDCs and macrophages exposed to total olive pollen lipids showed increased CD1d surface expression which resulted in the strong activation of iNKT cells	Enhances allergic sensitisation
Gilles	2009	Birch pollen	E1 phytoprostan-es (PPE1)	Dendritic cells (DCs)	Human	Analysed the role of PPE1 in regulating DC function and analysed its effect on NF-kappa-B signalling. DC phenotype was measured by flow cytometry and cytokine release by ELISA	PPE1 enhanced Th2 polarisation by modulating DC function *via* PPAR dependent pathways which inhibited NF kappa B activation, thus reducing DC IL-12 production	Enhances allergic sensitisation
Gilles	2010	Birch pollen	Aqueous birch pollen extracts, PPE1	Dendritic cells (slanDCs), T cells	Human	SlanDCs were stimulated with aqueous birch pollen extracts, with or without lipopolysaccharide (LPS). DC phenotype was measured by flow cytometry and cytokine release by ELISA.	PPE1 inhibited secretion of LPS-produced IL-12 p70 and IL-6. SlanDCs exposed to aqueous pollen extracts were impaired in eliciting an IFN-gamma response in naive CD4^+^ T cells	Enhances allergic sensitisation
Oeder	2015	Ragweed, birch, grass, or pine pollen	Aqueous pollen extracts (APEs), PPE1	B Cells	Murine and Human	B cells from murine splenocytes and from blood samples of healthy donors were incubated under Th2-like conditions with APEs or its constituents. Secreted total IgE was quantified by ELISA. B cell proliferation was measured by CFSE staining	PPE1 and Pollen extracts from various plant species enhanced Th2-induced production of total IgE and priming of B cells	Enhances allergic sensitisation
Gonzalez	2019	Timothy grass pollen	Aqueous pollen extracts PALMs	Dendritic cells	Murine	Bone marrow-derived DCs (BMDCs) were analysed by flow cytometry for changes in the expression of surface CD1d, in response APE stimulation. CD1d−/− BMDCs were used to rule out non-specific CD1d staining	Surface expression of CD1d on BMDCs was significantly increased in APE stimulated BMDCs	Enhances allergic sensitisation
Gutermuth	2007	Ovalbumin	Bet APE, PPE1	Dendritic cells	Murine	*In vitro* T cell responses to ovalbumin were measured or *in vivo* ova-specific CD4 T cells were transferred into mice. Mice were then challenged with ovalbumin with or without the presence of Bet APE/PPE1. Cytokines measured by ELISA.	PPE1 Inhibited LPS-induced IL-12p70 production of DCs. Bet APEs with allergen increased Th2 differentiation, whereas PPE1 and PPF1 inhibited TH2 proliferation and cytokine release	Enhances and inhibits allergic sensitisation
Bansal	2016	Cockroach extract	Lysophosphati-dylcholine (LPC)	NKT cells	Murine	Mice were sensitised to cockroach extract and LPC production was blocked by sPLA2. Anti-CD1d was also used to block CD1d. Bronchoalveolar lavage fluid (BALF) was collected and cytokine release measured by ELISA. Flow cytometry identified NKT populations	Cockroach extract activated phospholipids which secrete LPC. sPLA2 inhibition blocked LPC production which inhibited CD1d-restricted NKT cell activation. IL-4 and IL-5 secretion was blocked when LPC was inhibited	Enhances allergic sensitisation
Satitsuksanoa	2016	Der p 13	Fatty acid	Epithelial cells	Human	rDer p 13 ligand binding capacity was analysed by fluorescence-based lipid-binding assays, and *in silico* structural prediction. Cytokine release by sandwich ELISAs and epithelial activation assays were conducted	Der p 13 contained a potential binding site highly selective for hydrophobic ligands and can bind fatty acids. It triggered IL-8 and GM-CSF secretion in respiratory epithelial cells through a TLR2-, MyD88-, NF-kB-, and MAPK-dependent signalling pathway	Enhances allergic sensitisation

Key details of each aeroallergy study are presented, along with whether the study provides evidence for the role of lipids driving or inhibiting allergic sensitization.

The nine papers exploring the role of aeroallergen source-derived lipids can be grouped into three main mechanisms, two of which are similar to the food allergen studies; the activation of iNKT cells through CD1d molecules, and the direct activation of immune cells. The final mechanism reported was the activation of TLRs.

### CD1d-iNKT Cell Activation

As shown above with food allergies, lipids associated with aeroallergens have also been shown to influence allergic sensitization *via* CD1d-restricted iNKT cell activation. Four of the nine aeroallergy studies described lipids associated with aeroallergen sources were shown to be presented by CD1d molecules on APCs and subsequently activated iNKT cells (Agea et al., 2005; Bansal et al., 2016; Abos Gracia et al., 2017; González Roldán et al., 2019).

One study revealed PALMs primed DCs for the presentation of glycolipids to iNKT cells by CD1d upregulation (González Roldán et al., 2019). This supports findings from another study of olive pollen lipids (Abos Gracia et al., 2017), which established olive pollen lipids, but not aqueous pollen extracts (APEs), strongly activated human iNKT cells by increasing CD1d surface expression on iDCs and macrophages. All lipids analysed: polar lipids, diacylglycerols, free fatty acids, and triacylglycerol, were able to induce this increased CD1d expression. Despite altering the phenotype of iDCs, the olive pollen lipids did not alter their cytokine profile, but did induce secretion of IL-6 from macrophages, which further activated iNKT cells. Another study also found cypress pollen lipids were recognised by CD1d molecules (Agea et al., 2005). Furthermore, one study on cockroach allergy found the cockroach extract stimulated phospholipids to release lysophosphatidylcholine (LPC) and activate murine NKT cells, resulting in a Th2 shift. This NKT cell activation by LPC was inhibited when an anti-CD1d antibody was added (Bansal et al., 2016).

Overall, all four studies report lipids do promote allergic sensitization by presentation on CD1d molecules and activation of iNKT cells. Two of these studies scored 0.64 (Bansal et al., 2016) and 0.93 (Agea et al., 2005), with the latter scoring the highest in this review (Table 6). This study utilised a larger sample size, involving a well-defined human cohort. However, another two were some of the lower scores of this review studies; 0.43 (González Roldán et al., 2019) and 0.50 (Abos Gracia et al., 2017), primarily losing points due to lack of reporting sample features such as sample size, age of the participants, and defining their healthy and allergic cohorts.

**TABLE 6:** A summary of the quality of each aeroallergy study included in this systematic review.

First author (Year) [reference]	Sample quality			Methodological quality				Overall quality score (n/1)
	Sample size (n/2)	Defined controls (n/1)	Representative sample (n/3)	Model (n/2)	Robustness of model (n/2)	Lipid preparation (n/2)	Lipid characterisation (n/2)	
Abos Gracia (2017)	Unknown (0)	Yes (1)	Unknown (0)	Human (2)	Unknown (0)	Lipid extracted from olive pollen grains and purified. (2)	Polar lipids, diacylglycerols, free fatty acids, triacylglycerols (2)	0.50
Agea (2005)	14 (1)	Yes (1)	Six males, eight females, 19–45 years olds, allergic subjects (3)	Human (2)	Allergic subjects defined by clinical history of rhinoconjunctivitis and/or asthma, as well as positive skin prick tests and serum specific IgE levels (2)	Phospholipids commercially sought and prepared in absolute ethanol	Phospholipids: PC, PE (2)	0.93
						And lipids extracted from cypress pollen and purified. (2)		
Bansal (2016)	Unknown (0)	Yes (1)	Female BALB/c mice, 4–6 weeks old, allergic subjects (2)	Murine (1)	Mice sensitised by intraperitoneal (i.p.) injection (1)	LPC commercially sought (2)	LPC (2)	0.64
Gilles (2009)	Unknown (0)	Yes (1)	18–46 years olds, NO allergic subjects (1)	Human (2)	Healthy volunteers were screened for IgE against common allergens, and refrained from medication 2 weeks prior to blood sampling. (2)	Phytoprostanes extracted and purified from linoleic acid. (2)	Phytoprostanes PPE1 (2)	0.64
Gilles (2010)	Unknown (0)	Yes (1)	20–51 years olds, allergic subjects (2)	Human (2)	All subjects defined by total IgE serum levels. Allergic subjects had positive IgE against allergen, and a positive history of allergic rhinitis. All subjects refrained from medication for 15 days before blood donation. (2)	Phytoprostanes extracted and purified from linoleic acid. (2)	Phytoprostanes PPE1 and PPF1 (2)	0.79
González Roldán et al. (2019)	Unknown (0)	Yes (1)	Unknown, no allergic subjects (0)	Murine (1)	Unknown (0)	PALMs extracted from APEs and filtered/purified. (2)	APE, PALMs PPE1 and PPF1 (2)	0.43
Gutermuth (2007)	Unknown (0)	Yes (1)	Unknown (0)	Murine (1)	Unknown (0)	Phytoprostanes extracted and purified from linoleic acid	Bet-APE, PPE1 and PPF1 (2)	0.43
						APEs filtered from pollen grains.(2)		
Oeder et al. (2015)	Unknown (0)	Yes (1)	Female C57BL/6 and BALB/c mice, 6–10 week-old, allergic participants (2)	Human and Murine (2)	Mice sensitised by i.p. injection and total IgE measured (not allergen-specific IgE) (1)	Pollen grains commercially sought then filtered to obtain protein-free APEs. (2)	Amb-APE, PPE1 (2)	0.71
Satitsuksanoa (2016)	Unknown (0)	Yes (1)	Unknown (0)	Human (2)	Unknown (0)	Lipids commercially sought (2)	Cis-parinaric acid (2)	0.50

Studies were scored out of one for sample quality and methodological quality. Only aspects of each study relevant to the role of lipids in allergic sensitization were scored.

### Lipids Activate TLRs

One of the nine aeroallergen studies investigated the lipid activation of Toll like receptors (TLRs) (Satitsuksanoa et al., 2016). This study suggests the HDM protein allergen, Der p 13, has certain structural features, allowing highly selective lipid-binding of fatty acids.

The protein-lipid complex can then activate TLRs, such as TLR2, to stimulate inflammatory cytokines IL-8 and GM-CSF production in respiratory epithelial cells. This study concludes lipids enhance allergic sensitization, however, the study was awarded a below-average quality score of 0.50 (Satitsuksanoa et al., 2016) (Table 6). Again, this was mostly due to a lack of reporting sample sizes and not defining their cohorts.

### Immune Cell Activation

All five studies reporting aeroallergen lipids can directly activate immune cells focused on PALMs (Agea et al., 2005; Gutermuth et al., 2007; Gilles et al., 2009; Gilles et al., 2010; Oeder et al., 2015). Two human studies (Gilles et al., 2009; Gilles et al., 2010) highlighted aqueous birch pollen extracts (Bet.-APE)-derived PPE_1_ modulated DC function and its cytokine production, specifically the inhibition of IL-12, preferentially inducing a Th2 response. Another study using a murine model of allergy found PPE_1_ inhibited the LPS-induced production of IL-12 from DCs (Gutermuth et al., 2007), but when intranasally instilled with the egg allergen protein, Ovalbumin, PPE_1_ inhibited Th2 polarization and cytokine release, suggesting lipids inhibit allergic sensitization. Another study also established PPE_1_ and aqueous pollen extracts stimulated Th2-primed B cells to enhance IgE production (Oeder et al., 2015). One study, previously mentioned for its evidence of CD1d recognition of PALMs, found the presence of PC and PE lipids from cypress pollen alone stimulated TCR αβ^+^ CD4^+^ T cell production of IL-4, enhancing a Th2 response (Agea et al., 2005).

Overall, four studies show PALMs can directly activate DCs, B cells, and CD4^+^ T cells to shift to a Th2 response. Whereas, one study (Gutermuth et al., 2007) reports PALMs can promote and inhibit allergic sensitization. The quality assessment (Table 6) highlights three of the studies which suggested lipids promote allergic sensitization were above average; 0.71 (Oeder et al., 2015), 0.79 (Gilles et al., 2010), and 0.93 (Agea et al., 2005). However, one study (Gilles et al., 2009), as well as the study reporting lipids both promote and inhibit allergic sensitization (Gutermuth et al., 2007), scored below average; 0.64 (Gilles et al., 2009) and 0.43 (Gutermuth et al., 2007). As before, this was mostly due to lack of reporting sample sizes, how representative the sample was, and how robust the model was.

### Summary of Lipids in Aeroallergies

The mechanisms proposed include aeroallergens proteins can bind to lipids and activate TLRs to shift to a Th2 response. PALMs, notably PPE_1,_ can act upon DCs to modulate its subsequent cytokine release to favour allergic sensitization. Similar to food allergenic lipids, aeroallergen lipids can also activate iNKT cells through CD1d presentation.

## Discussion

**FIGURE 2:**
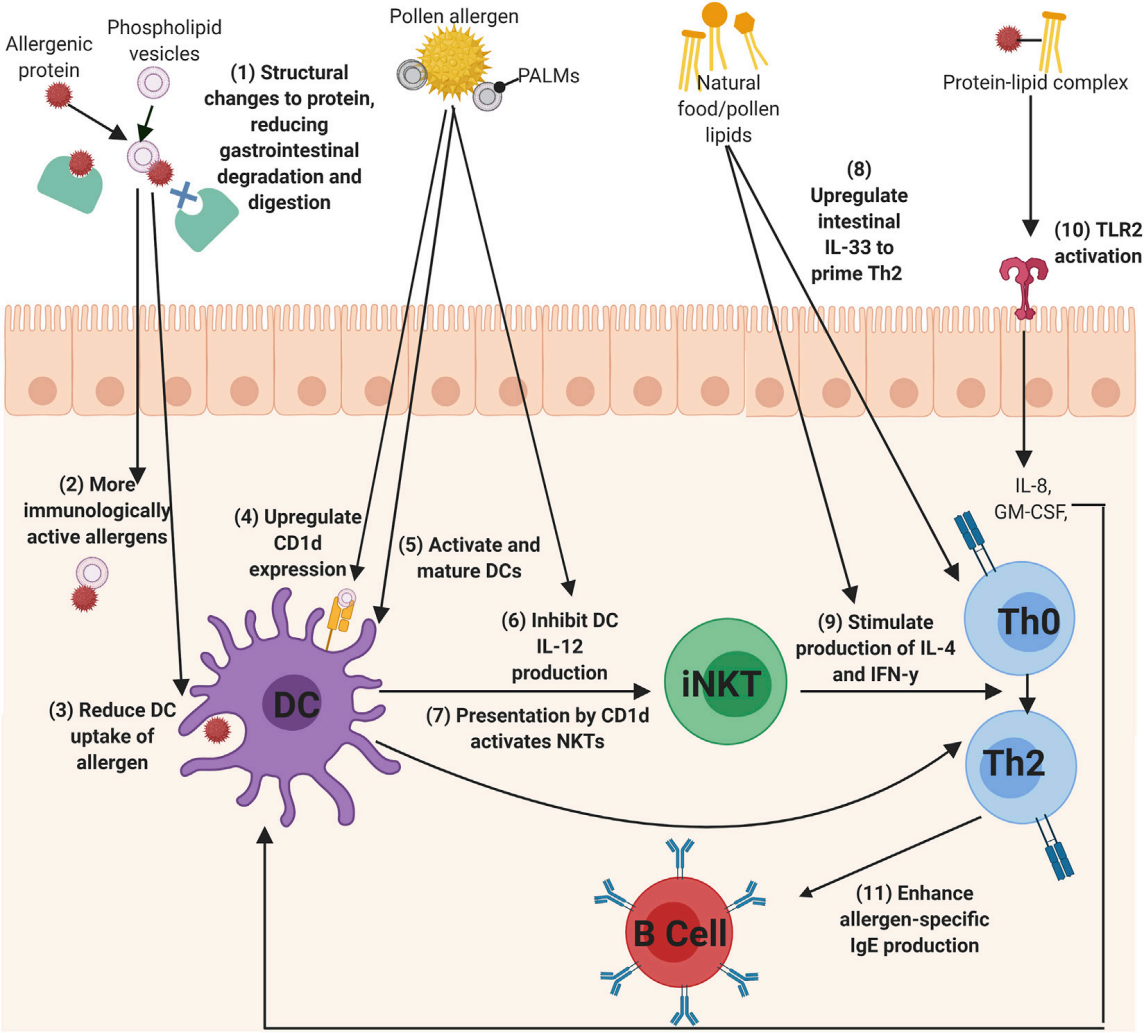
The mechanisms of lipids to influence allergic sensitization. (1) Phospholipids can bind allergens to reduced gastrointestinal degradation of the allergen, which (2) allows more immunologically active allergens to enter the immune system and can also (3) alter DC uptake of the allergen. Lipids, such as PALMs, can directly act upon DCs by (4) upregulating CD1d expression, (5) activating and maturing DCs, (6) and inhibiting I-12 production, which can all lead to the (7) activation of iNKT cells. Th0 cells could then be primed to Th2 cells by (8) IL-33 secretion from lipid-activated epithelial cells, or (9) by the secretion of IL-4 and IFN-y cytokines from lipid-activated iNKT cells. (10) protein-lipid complexes can activate TLRs, such as TLR2, to initiate IL-8 and GM-CSF production, which in turn activates DCs. Finally, (11) lipids can also enhance the production of allergen-specific IgE from B cells (Created using Biorender.com).

Allergens from food, pollen, and insect faecal particles are delivered to the immune system in association with a variety of immunomodulatory components, such as lipids. This systematic review captures growing evidence for the role of lipids in allergic sensitization. In summary, lipids can interact with allergenic proteins to influence the development of allergic sensitization. This protein-lipid interaction resulted in reduced gastrointestinal degradation of the allergenic proteins through structural protein changes, the reduction of DC uptake of allergenic proteins to reduce immune tolerance, the regulation of Th2 cytokines, the enhancement of allergen-specific IgE, the activation of iNKT cells through CD1d ligation, and finally, directly acting upon TLRs, epithelial cells, keratinocytes, and DCs. Figure 2 summarises the main mechanisms identified in this review of how lipids influence allergic sensitization.

The results of this systematic review show 18 out of 19 (Agea et al., 2005; Dearman et al., 2007; Gilles et al., 2009; Gilles et al., 2010; Jyonouchi et al., 2011; Mirotti et al., 2013; Oeder et al., 2015; Angelina et al., 2016; Bansal et al., 2016; Satitsuksanoa et al., 2016; Abos Gracia et al., 2017; Tordesillas et al., 2017; Pablos-Tanarro et al., 2018; Palladino et al., 2018; González Roldán et al., 2019; Finkina et al., 2020; Meng et al., 2020) studies reported lipids can enhance allergic sensitization, revealing a strong weight of evidence towards the role of lipids in driving a Th2-type response. Although, two of these studies also report lipids can inhibit allergic sensitization (Gutermuth et al., 2007; Palladino et al., 2018). And one paper in this review solely suggests lipids inhibit allergic sensitization (Hufnagl et al., 2018).

For the 95% of studies in the systematic review reporting lipids can enhance allergic sensitization, a key finding was the ability for lipids intrinsic to food and inhalant allergen sources to promote allergic sensitisation *via* the activation of CD1d-restricted iNKT cells, with 47% of studies reporting this mechanism. During allergic sensitization, in contrast to proteins that are presented by MHC class II molecules, it is well-established that lipids are presented by CD1 molecules (Schiefner and Wilson, 2009). There has been particular interest in CD1d molecules as they present glycolipids to a specific group of T lymphocytes, called invariant natural killer T (iNKT) cells, which are powerful immune regulators (Dhodapkar and Kumar, 2017). This data supports existing knowledge of the presentation of lipids by CD1d molecules on APCs, and the subsequent recognition by iNKT cells, to result in the release of Th2-skewing cytokines. As all eight studies which reported this mechanism enhances allergic sensitization, it provides a strong weight of evidence for CD1d-restricted iNKT cells in the role of enhancing, or in some cases initiating, allergic sensitization. On another note, not all lipids tested were successful in activating iNKT cells, which potentially indicates a specificity for iNKT cells to recognise CD1d-lipid complexes. Some research suggests this specificity could be due to the structure of the lipid, specifically its head group, as self-lipids with larger head groups decreased or prevented interaction with iNKT-TCRs (Mallevaey et al., 2011).

Another key mechanism was the ability for lipids to directly and indirectly activate cells of the immune system, such as DCs, NKT cells, T cells, keratinocytes, epithelial cells and B cells, with 53% of studies reporting this mechanism. The studies highlighted the ability for certain food allergen-associated lipids to reduce human moDC protein uptake (Angelina et al., 2016). High doses of protein uptake leads to the induction of tolerance, whereas low doses of allergenic protein uptake favours a Th2 response (Wisniewski et al., 2013). Hence the reduction of Sin a 2 and Ara h 1 capture by human moDCs reduced the allergen tolerance and thus shifted to/enhanced the development of allergic sensitization. Interestingly, the administration of peanut lipids accompanied with peanut allergenic protein inhibited the production of the anti-inflammatory cytokine, IL-10, from keratinocytes (Palladino et al., 2018). Thus, the co-delivery of peanut lipids with peanut protein promotes an inflammatory state, favouring a Th2 response. Aeroallergen studies focused on the role of PALMs in allergic sensitization. PALMs are hypothesised to induce and enhance allergic sensitization. Pollen grains co-release allergenic proteins and PALMs when stimulated with water, which can then interact to form protein-lipid complexes (Bashir et al., 2013). Once released by the pollen grain, PALMs can then interact with pollen-exposed human epithelia (Gilles et al., 2009). The five studies investigating PALMs explored their ability to act upon DCs, B cells and T cells. For instance, one study found PALMs induced CD1d upregulation on murine DCs. This is likely a result of preparing the cell for lipid presentation to iNKT cells, which once activated, can release Th2 cytokines (González Roldán et al., 2019). Similarly, another study using a murine model of allergy found the PALM, PPE_1,_ inhibited the LPS-induced production of IL-12 from DCs (Gutermuth et al., 2007). Importantly, IL-12 production promotes a Th1 response, and in its absence, a Th2 response is favoured (Moser and Murphy, 2000). Thus, this explains why the inhibition of IL-12 noted in three studies resulted in a Th2 shift.

Four studies suggested lipids can act as an adjuvant to IgE production during allergic sensitization. The PALM, PPE_1,_ is the main lipid component of birch and ambrosia APEs (González Roldán et al., 2019). PPE_1_ is structurally similar to endogenous prostaglandins, which have also been reported to stimulate IgE production from B cells (Roper et al., 1990), hence this could explain the structure of PALMs determines its adjuvant activity. Lipids administered alone shifted towards a Th2 reaction, (Palladino et al., 2018). This is similar to another study who found the lipid fraction of the Brazil nut allergenic protein, Ber e 1, was essential to stimulate an IgE response (Mirotti et al., 2013). Another study found Brazil nut sterols and polar lipids all had marked adjuvant effects on IgE production. However, other Brazil nut lipids, β-sitosterol and glycolipid-rich fractions, did not impact IgE (Dearman et al., 2007). Thus, it is important to note that there is some specificity for lipids driving allergic sensitization, potentially determined by structural qualities.

Another mechanism, proposed by one aeroallergy study, suggested the Der p 13 lipid-ligand can activate TLR2 to stimulate inflammatory cytokines in epithelial cells (Satitsuksanoa et al., 2016). This was the only paper in the systematic review stating this lipid-induced effect. Although, this mechanism is endorsed by a recent review which explained other lipids, such as membrane-bound lipids, can also influence TLR activity (Ruysschaert and Lonez, 2015).

The final mechanism reported in this systematic review regarding lipids enhancing allergic sensitization was lipid-induced conformational changes to allergenic proteins, which enhanced allergenicity. One study highlighting this mechanism found phospholipid-binding resulted in reduced gastrointestinal degradation of the peanut allergenic protein (Angelina et al., 2016). This resistance to degradation allows immunologically active protein allergens to reach the gut immune system and trigger allergic sensitization by presentation to DCs, and also trigger the effector phase upon further exposure. Another two studies investigating the lipid-protein binding of lentil allergens (Finkina et al., 2020) and milk allergens (Meng et al., 2020) supported these findings, adding that lipid-binding enhances thermostability of allergenic proteins during digestion. In contrast, this was not the case in the mustard seed allergenic protein, Sin a 3, which is structurally different to peanut allergen proteins, and was not affected by the presence of PG vesicles (Angelina et al., 2016). As aforementioned, this infers allergen structure may determine the interaction with lipid membranes, affecting DC uptake of the protein. Overall, the ability for lipids to favour allergic sensitization through altering the structure of its associated allergenic proteins is well supported. However, it must be noted that different lipids have different effects on the secondary structures of allergenic proteins, even those proteins which are from the same class and structurally similar. A recent review on protein-lipid binding supports lipids in inducing conformational changes to the allergenic protein and the subsequent altered allergenic properties, as well as highlights the different structural effects induced by different lipids (Jappe et al., 2019).

The three studies with reports that lipids can inhibit the development of allergic sensitization investigated peanut lipids (Palladino et al., 2018), retinoic acid from milk (Hufnagl et al., 2018), and PPE_1_ from birch pollen (Gutermuth et al., 2007). Although, the study of peanut lipids had the third lowest quality score of all 19 studies, thus, the findings should be interpreted with caution. In contrast, the study into the role of retinoic acid in allergic sensitisation was awarded a higher than average quality score of 0.86. The proposed mechanisms of lipid-inhibited allergic sensitization include the inhibition of Th2 cytokine secretion (Gutermuth et al., 2007; Hufnagl et al., 2018) and the upregulation of Th1 cytokine secretion (Palladino et al., 2018). The study that solely states lipids inhibit allergic sensitization reported that stimulation with retinoic acid bound to Bos d five milk allergenic protein supressed IL-10 and IL-13 cytokine release. Thus, this study suggests Bos d five loading of retinoic acid supresses a Th2 response and its allergenicity. This Bos d five loading of retinoic acid correlated with reduced lysosomal digestion of the protein allergen. Despite this study inferring some lipids do not promote allergic sensitization, it still provided clear evidence for the formation of protein allergen-lipid binding, which is a key phenomenon that highlights allergenic proteins are co-delivered to the immune system with other compounds, such as lipids. The study on birch pollen allergy also reported lipids can inhibit allergic sensitization when the lipid PPE_1_ was delivered with an allergenic protein, as its complex inhibited Th2 polarization and cytokine release (Gutermuth et al., 2007). Again, suggesting lipids inhibit allergic sensitization when accompanied by its associated protein allergen. The findings of these two studies contrasts with another study reporting lipids can inhibit allergic sensitization, as it stated the accompaniment of peanut lipids without its associated allergenic protein actually stimulated Th1 cytokines, IL-10, to be released (Palladino et al., 2018), and suggesting lipids can inhibit allergic sensitization alone. Overall, the three studies stating lipids can inhibit allergic sensitization put importance on the effect of delivering lipids accompanied by allergenic proteins to the immune system, with two studies implying lipids co-delivered with allergenic proteins inhibits a Th2 response, and one study contrasting to state lipids without allergenic proteins actually inhibit a Th2 response.

A major criticism of the studies examined is the quality assessment scores, as many of the included studies were low. The aim of this scoring system was to highlight the robustness of existing research in this area. Low-scoring studies are not to be deemed unreliable, but reflect the need for further research which has adopted specific characteristics, such as larger sample sizes and human model systems. The majority of these studies lost points due to lack of reporting sample sizes and defining the cohort of samples. Indeed, only 53% of studies published data on sample size. Of this data, only two studies (Jyonouchi et al., 2011; Hufnagl et al., 2018) employed a cohort of above 20. This limits the power of the study to detect associations and highlights the need for more studies to report sample sizes. Furthermore, no statistical power calculations to determine sample size were evident throughout the papers.

In contrast, all studies (excluding one (Pablos-Tanarro et al., 2018)) received high scores for the preparation of their lipid, where they either commercially sought lipids or provided detailed methods for the extraction and purification of lipids. Most studies also scored highly for characterisation of the lipid used. Although, several studies lacked clarification on the type of lipids they utilised (Angelina et al., 2016; Pablos-Tanarro et al., 2018; Palladino et al., 2018), simply stating “peanut lipids” or “egg lipids.” The range of lipids encapsulated in an allergen source is wide, thus, the lack of specificity then poses difficulty in drawing conclusions to which lipid promotes or inhibits a Th2 response.

This systematic review included studies utilising human and murine models, with 47% of the studies using murine models of allergic sensitization. The use of mice to study allergic sensitization could be deemed important in addition to human data. However, studies solely recruiting murine models needs to be cautiously interpreted, taking into consideration differences to the human immune system and a lack of validated animal models. For instance, mice only express CD1d receptors on DCs, and cytokines, such as IL-10, produced by a Th2 response in mice are produced by a Th1 and Th2 response in humans (Mestas and Hughes, 2004). Furthermore, allergy in mice is not natural, thus, inducing sensitization to allergens is artificial and does not fully reflect the development of allergic sensitisation in humans. Based on this information, murine models were scored lower in the quality assessment due to the potentially reduced human relevance.

General limitations of this systematic review include the lack of evaluation for differences in lipid metabolism between males and females. It is evident that gender was not considered in most studies, but evidence suggests the lipid metabolism differences between genders could lead to differences in immune responses (Furman et al., 2014). Thus, future studies should consider this factor. Furthermore, during the article search stage of this systematic, there were many relevant papers excluded from this review as they were conference abstracts, rather than peer-reviewed publications. For instance, there was a collection of studies relevant to non-specific lipid transfer proteins (nsLTPs), but these were only available as conference abstracts (Smole et al., 2011; Gepp et al., 2014; Gilles et al., 2014; Palladino et al., 2016; Garrido-Arandia et al., 2018; Iweala et al., 2018; Foo et al., 2019; Humeniuk et al., 2019; Perez Rodriguez et al., 2019). Once, or if, these conference abstracts have been published as articles, an updated review would be beneficial.

Future research of lipids in allergic sensitization could allow pathomechanistic insights, leading to the development of new treatments and prevention approaches to type 1 hypersensitivity, during this allergy epidemic. It is evident that the effect lipids have on allergic sensitization differs depending on the lipid and protein class. Thus, future research is needed to identify the specific lipids involved in enhancing Th2 pathways, and to characterize their potential interaction with allergenic proteins. Ultimately, this research highlights that it is the combination of components from the allergen source which promote allergic sensitization. Hence, it is key that these components are studied together and their combined effects on the immune system measured.

This research also highlights the importance of whole allergen source extracts used in allergy diagnostics, such as skin-prick testing. As using purified allergenic proteins, without the lipid cargo present, may result in false-negative responses, due to some research suggesting the lipid fraction must be present in order to trigger a Th2 response. Furthermore, establishing factors which enhance allergic sensitization are essential to identify potential food allergens as part of food safety assessment processes. Especially as novel foods are constantly being introduced to consumers to counteract the food insecurity problem, the importance of assessing the allergenicity of food proteins in key. The incorporation of lipids into current immunogenicity assays could therefore provide critical evidence to the assessment of protein allergenicity.

Ultimately, this systematic review concludes lipids intrinsic to an allergen source can act as immune adjuvants, through the various mechanisms discussed. Adjuvants are defined as substances which have the capacity to enhance the immune response to an allergen (Berin and Shreffler, 2008). Thus, it could be speculated that the allergenicity of protein allergens could be determined by the presence of the lipids. However, due to the limited number of papers available for this systematic review, further research is essential to validate these findings, before the results can be applied elsewhere. Overall, there is a consensus that lipids do promote allergic sensitization.

## Data Availability

The original contributions presented in the study are included in the article/Supplementary Material, further inquiries can be directed to the corresponding author.

## Abbreviations

APE, Aqueous pollen extract; BEH, Behenic acid; BLA, α-lactalbumin; BLG, β-lactoglobulin; CD1, Cluster of differentiation 1; DC, Dendritic cell; Df, *Dermatophagoides farina*; ELISA, Enzyme-linked immunosorbent assay; GM-CSF, Granulocyte-macrophage colony-stimulating factor; HDM, House dust mite; hmoDC, Human Monocyte-derived dendritic cell; IFN, γ Interferon gamma; IgE, Immunoglobulin E; IL, Interleukin; iNKT, invariant natural killer T cells; LAU, Lauric acid; LPC, Lysophosphatidylcholine; LPS, Bacterial lipopolysaccharide; MHC, Major Histocompatibility Complex; nsLTP, Non-specific lipid-transfer protein; OLE, Oleic acid; PALM, Pollen-associated lipid mediator; PG, Phosphatidylglycerol; PPE_1_, Phytoprostanes E1; STE, Stearic acid; Th1, T cell subset 1; Th2, T cell subset 2; TLR, Toll-like receptor; UFA, Unsaturated fatty acids.
